# Whole-genome sequencing reveals that mutations in myosin-5 confer resistance to the fungicide phenamacril in *Fusarium graminearum*

**DOI:** 10.1038/srep08248

**Published:** 2015-02-04

**Authors:** Zhitian Zheng, Yiping Hou, Yiqiang Cai, Yu Zhang, Yanjun Li, Mingguo Zhou

**Affiliations:** 1 College of Plant Protection, Nanjing Agricultural University, Key Laboratory of Pesticide, Jiangsu Province, Nanjing, 210095, China

## Abstract

To determine the mechanism of resistance to the fungicide phenamacril (JS399-19) in *Fusarium graminearum*, the causal agent of Fusarium head blight, we sequenced and annotated the genome of the resistant strain YP-1 (generated by treating the *F. graminearum* reference strain PH-1 with phenamacril). Of 1.4 million total reads from an Illumina-based paired-end sequencing assay, 92.80% were aligned to the *F. graminearum* reference genome. Compared with strain PH-1, strain YP-1 contained 1,989 single-nucleotide polymorphisms that led to amino acid mutations in 132 genes. We sequenced 22 functional annotated genes of another *F. graminearum* sensitive strain (strain 2021) and corresponding resistant strains. The only mutation common to all of the resistant mutants occurred in the gene encoding myosin-5 (point mutations at codon 216, 217, 418, 420, or 786). To confirm whether the mutations in myosin-5 confer resistance to phenamacril, we exchanged the myosin-5 locus between the sensitive strain 2021 and the resistant strain Y2021A by homologous double exchange. The transformed mutants with a copy of the resistant fragment exhibited resistance to phenamacril, and the transformed mutant with a copy of the sensitive fragment exhibited sensitivity to phenamacril. These results indicate that mutations in myosin-5 confers resistance to phenamacril in *F. graminearum*.

Phenamacril (experimental code JS399-19; a.i. 2-cyano-3-amino-3-phenylancryic acetate), which was discovered and patented by the Jiangsu Branch of the National Pesticide Research and Development South Center of China, is a cyanoacrylate fungicide. Cyanoacrylate compounds have a novel mode of action and are considered to be environmentally safe^1^. Many cyanoacrylates that contain heterocycles exhibit excellent activity against weeds^2^, insect pests, fungal pathogens^3^, viruses^4^, and cancer^5^. One of these, phenamacril, is a *Fusarium*-specific fungicide that is used to control Fusarium head blight (FHB). FHB is a devastating disease of cereal crops worldwide^6^,^7^. In addition to reducing grain yield and quality, the causal agent, the fungus *Fusarium graminearum* (teleomorph *Gibberella zeae*), produces harmful mycotoxins in infected grain^8^,^9^. The most efficient strategy for the control of FHB is through the application of fungicides during wheat anthesis. The use of phenamacril reduced both the FHB index and mycotoxin level by 80%^10^,^11^,^12^.

Although phenamacril strongly inhibits the mycelial growth of *F. graminearum* and can provide excellent control of FHB in the field^10^, the fungus has frequently developed resistance to phenamacril under selection pressure from the fungicide in laboratory trials^11^,^13^. Elucidating the resistance mechanism of *F. graminearum* to phenamacril is important because it will increase our understanding of the fungicide's mode of action and of ways to avoid the development of resistance.

The molecular mechanisms of phenamacril toxicity in *F. graminearum* are still unknown. According to the results of our previous studies, resistance to phenamacril in *F. graminearum* is governed by a single major gene, and there is no cross-resistance between phenamacril and well-known fungicides belonging to other chemical classes, such as ergosterol biosynthesis inhibitors (tebuconazole and prochloraz) and strobilurins (azoxystrobin), suggesting that the mode of action and resistance mechanisms of phenamacril are different from those of other fungicides^10^,^13^,^14^. However, we identified fimbrin as a key protein regulating resistance to phenamacril in our previous study; disruption of the FgFim gene in both a resistant strain (Y2021A) and a sensitive strain (2021) significantly reduced the EC_50_ values^15^. A susceptible phenotype theoretically indicates that the deleted gene is important for conferring drug resistance in the wild-type strain^16^,^17^. Based on the integration of chemical-genetic and genetic interaction data^18^ for phenamacril and *F. graminearum*, we inferred that there was close relationship between resistant target pathways or proteins and the actin skeleton^15^.

Myosins are eukaryotic, actin-dependent ATPase motors that play important roles in cytokinesis, actin filament bundle organization, vesicle/organelle transport, cell polarization, transcriptional regulation, intracellular transport, and signal transduction^19^,^20^,^21^,^22^. Almost all myosins travel toward the barbed (+) ends of actin microfilaments^23^,^24^. Based on genomic survey and phylogenetic analyses, 31 myosin classes have been defined^25^. In humans, 39 genes encode for myosin, and these are organized into 12 classes^26^. The budding yeast (*Saccharomyces cerevisiae*) has one type II myosin (Myo1p), two type I myosins (Myo3p and Myo5p), and two type V myosins (Myo2p and Myo4p). Type I myosins (Myo3p and Myo5p) localize to endocytic sites and are essential for endocytic internalization^27^,^28^,^29^. The type II myosin (myo1p) is involved in the contraction of the actin ring in cytokinesis^30^. Type V myosins (Myo2p and Myo4p) transport a variety of cargoes along actin cables. In *F. graminearum*, the myosin gene family has three members, including FGSG_08719.1^31^, which encodes an uncharacterized protein; FGSG_07469.1, which encodes myosin-2B; and FGSG_01410.1, which encodes myosin-5. All three of these myosin proteins have conserved “head” regions. The head or motor domain contains binding sites for ATP and actin. The myosin-2B protein has an isoleucine- and glutamine-rich IQ motif, which are possible binding sites for calmodulin or calmodulin-like light chains. The myosin-5 protein has an src homology 3 (SH3) domain, which might mediate protein–protein interactions that regulate enzymatic activity^32^.

Complete genome sequences of several pathogenic bacteria have recently been determined. Even though microbial genomics has had little direct impact on antibacterial drug discovery so far, the possibilities of using complete genome sequences for target identification are virtually unlimited^33^. In this paper, we use whole-genome sequencing to confirm that mutations in myosin-5 are the cause of phenamacril resistance in *F. graminearum*.

## Results

### Whole-genome sequencing statistics

To identify genes that might contribute to phenamacril resistance or to the cytotoxic action of this fungicide, we sequenced and annotated the genome of the resistant strain YP-1. The basic whole-genome sequencing statistics are shown in Table 1. The sequencing depth was 115×, and read mapping results indicated nearly complete genome coverage (~99.35%) for the strain. Compared with reference strain PH-1, a total of 262 mutations (excluding the synonymous mutations) in coding sequences (CDS) were identified in strain YP-1, which lead to amino acid mutations for 132 genes (see Supplementary Table S2 online). Among 1,989 single-nucleotide polymorphisms SNPs changes, the base changes were mainly A to G and T to C transition mutations, and the proportion were 16.9% and 16.9%, respectively (see Supplementary Fig. S1 and Table S3 online).

### Identification of the mutations in other resistant strains compared to the corresponding sensitive strain

**Figure 1:**
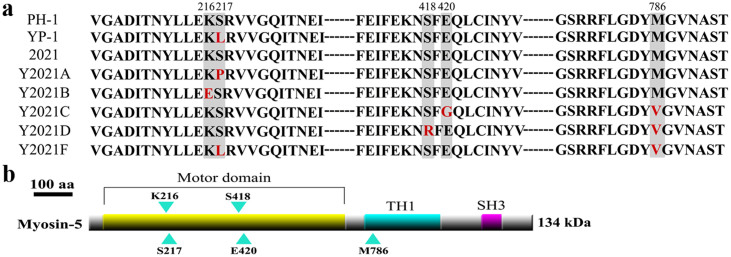
Phenamacril resistant mutants contain the point mutations at codon 216, 217, 418, 420 and 786 of myosin-5 in *Fusarium graminearum*. (a): Alignment of partial deduced amino acid sequences of myosin-5 from the reference strain PH-1, induced resistant strain YP-1 from PH-1, wild-type strain 2021 and induced resistant strains Y2021A, B, C, D, F from 2021. The vertical boxes indicate the amino acid changes at the codon 216, 217, 418, 420 and 786 that are responsible for phenamacril resistance. (b): Schematic representation of *Fusarium graminearum* myosin-5. Sites of lysine, serine, glutamic acid and methionine mutantions are indicated with blue arrowheads. The conserved motor domain, myosin tail (TH1) and src homology domain 3 (SH3) are highlighted.

Among the 132 genes that resulted in amino acid mutations, only 22 are known in the *F. graminearum* database (see Supplementary Table S2 online). First, we sequenced the 22 genes of the phenamacril-sensitive strain 2021 and of the corresponding phenamacril-resistant strains Y2021A, B, C, D, and F. Only myosin-5 (FGSG_01410) exhibited point mutations in all five resistant mutants compared with the phenamacril-sensitive strain 2021. Nucleotide changes occurred at positions 216, 217, 418, 420, or 786 in the predicted deduced amino acid sequence of the myosin-5 gene (Fig. 1a and Table 2). Phenamacril-sensitive strains PH-1 and 2021 had a lysine at position 216, a serine at positions 217 and 418, a glutamic acid at position 420, and a methionine at position 786. The six phenamacril-resistant strains had the following mutations: YP-1 had a leucine at codon 217; Y2021A had a proline at codon 217; Y2021B had a glutamic acid at codon 216; Y2021C had a glycine at codon 420 and a valine at codon 786; Y2021D had an arginine at codon 418 and a valine at codon 786; and Y2021F had a leucine at codon 217 and a valine at codon 786 (Fig. 1a and Table 2).

### Gene replacement strategy of myosin-5 between sensitive strain and resistant strain

**Figure 2:**
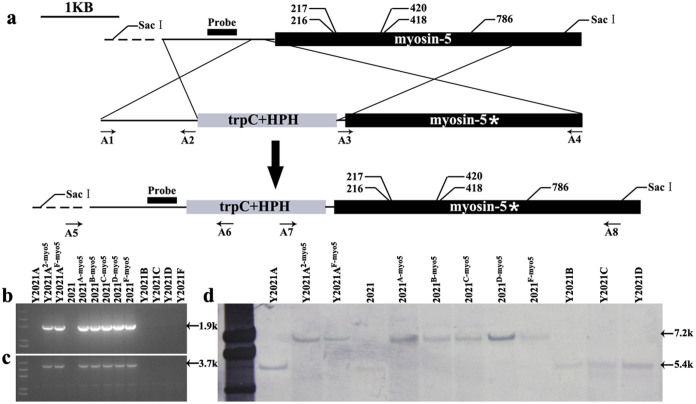
Generation and identification of *Fusarium graminearum* myosin-5 gene recombinant mutants by gene replacement. (a) Schematic representation of gene replacement strategy. The upper part represents the genomic locus target of the replacement construct. The black dashed lines represent the surrounding genomic region. The gene replacement cassette contains the hygromycin resistance gene and the myosin-5 mutated zone. Primer binding sites are indicated by arrows (see Table S1 for the primer sequences). The asterisk represents the selected myosin-5 mutated zone. (b) and (c) Polymerase chain reaction (PCR) strategy to screen replacement transformants: (b) PCR performed with primer pair A5/A6; a 1.9-kb amplified fragment indicates replacement integration at the left junction. (c) PCR performed with primer pair A7/A8; a 3.7-kb amplified fragment indicates replacement integration at the right junction. (d) Southern blot hybridization analysis of 2021 and all the mutants using a 622-bp myosin-5 upstream fragment as probe, and genomic DNA digested with SacI.

To further test whether the mutations in myosin-5 confer resistance to phenamacril, we replaced the myosin-5 locus between sensitive and resistant strains by homologous double exchange. Firstly, the myosin-5 loci of resistant strains Y2021A, B, C, D, and F were introduced individually into the sensitive strain 2021 by gene replacement^34^. Secondly, the myosin-5 of the sensitive strain 2021 and the resistant strain Y2021F were introduced individually into resistant strain Y2021A, whose resistance level differed from that of Y2021F. Briefly, the 2021 sensitive recipient strain and Y2021A resistant recipient strain were transformed with the replacement cassette described in Figure 2a. The gene replacement events were validated in the selected transformants by PCR and sequencing (Fig. 2b and c, and Table 2). Single copy insertions at the myosin-5 locus were verified by Southern blotting (Fig. 2d).

Sequence analysis of the six transformants confirmed that codons were altered as designed (Table 2), such as, in Y2021A-10^2-myo5^, codon 217 (CCA) for proline was altered to a codon for serine (TCA). In Y2021A-24^F-myo5^, codon 217 (CCA) for proline was altered to a codon for leucine (TTA), and codon 786 (ATG) for methionine was altered to a codon for valine (GTG).

### Sensitivity of the myosin-5 mutants to phenamacril

**Figure 3:**
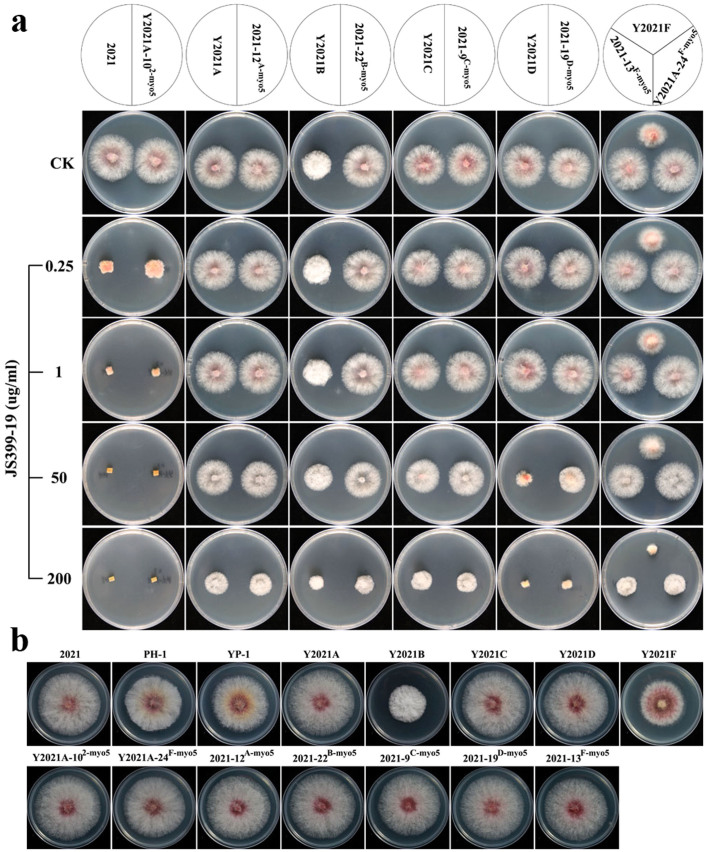
Colony morphology of *Fusarium graminearum* strains. (a): Effects of phenamacril (JS399-19) on mycelial linear growth of *F. graminearum* wild-type strain 2021, resistant strains Y2021A, B, C, D, F and myosin-5 replacement mutants on PDA. The mycelial plugs taken from the edge of a 3-day-old colony of strains or mutants were grown at 25°C for 2 days on the PDA plates, which were amended with JS399-19 at 0, 0.25, 1, 50, or 200 μg a.i./ml. (b): Colony morphology of 2021, PH-1 and all the mutants. Strains were grown on solid media (PDA) for 3 days at 25°C.

The susceptibility of the generated myosin-5 mutants to phenamacril was assessed based on mycelial growth^35^ in fungicide-amended and fungicide-free media at 25°C. All of the myosin-5 mutants were resistant to phenamacril (EC_50_>40), but their resistance levels differed (Table 3). Y2021D was moderately resistant (EC_50_ = 42), while YP-1 and Y2021F were highly resistant (EC_50_ = 146 and 149), which indicated that the mutation at codon 786 is not the primary mutation resulting in phenamacril resistant and may not even affect phenamacril resistance (Table 2). Y2021A, B, and C were highly resistant (EC_50_ >200). Furthermore, mutants transformed with a copy of resistant fragments exhibited resistance, and mutants transformed with a copy of the sensitive fragment exhibited sensitivity to phenamacril. The EC_50_ values in all transformed mutants were basically restored to the sensitivity phenotype of the strains that donated the replacement of the myosin-5 locus (Table 3). For example, Y2021A-10^2-myo5^, which had been transformed with the myosin-5 locus of strain 2021, could not grow on PDA containing 1 μg/ml JS399-19. However, all of the transformed mutants with the myosin-5 locus of resistant strains could grow on PDA containing >50 μg/ml JS399-19 (Fig. 3a).

In conclusion, all of the myosin-5 mutations identified in the transformed resistant strains confer resistance to phenamacril. Our results indicate that the resistance levels can be fully accounted for by mutations in the myosin-5 gene.

### Myosin-5 sequence and domain analysis

The complete DNA sequences of a myosin-5 locus (ranging from 313 bp upstream to 202 bp downstream of the myosin-5 coding region) from six strains of *F. graminearum* were obtained following PCR amplification using primers 01410F/01410R (see Supplementary Table S1 online). Myosin-5 has an open reading frame (ORF) of 3,645 bp interrupted by two introns. The ORF encodes a putative protein of 1214 amino acids, with a calculated molecular weight of 134 kDa. There were 19 nucleotide differences, in total, between the myosin-5 from the six Chinese strains and the sequenced reference strain PH-1.

In addition to a motor domain, myosin-5 has a myosin tail (TH1) domain and an SH3 domain (http://www.ncbi.nlm.nih.gov/protein/ESU06723.1). However, the amino acid mutations at codon 216, 217, 418, and 420 occur in the myosin motor domain, and only one mutation, at codon 786, occurs in the TH1 domain (Fig. 1b). Furthermore, the results presented in the previous section prove that the mutation at codon 786 does not affect phenamacril resistance.

In our previous study, we tested the antifungal properties of phenamacril against 12 economically important plant pathogens and found that phenamacril strongly inhibited the mycelial growth of *Fusarium* spp., such as *F. graminearum* and *Fusarium oxysporum*, but had little or no activity against other fungal pathogens, including *Botrytis cinerea*, *Pyricularia grisea* (*Magnaporthe oryzae*), and *Blumeria graminis*^10^. Because the mutations of the myosin-5 motor domain confer resistance to phenamacril in *F. graminearum*, we wondered whether the lack of sensitivity to phenamacril in the other pathogens was associated with a different myosin motor domain. Therefore, we aligned the homologous myosin motor domains of *F. graminearum*, *F. oxysporum*, *B. cinerea*, *M. oryzae*, and *B. graminis*. We found that the myosin-5 motor domain from *F. graminearum* shares 97.6% identity with the myosin1 (EWY80136.1) motor domain from *F. oxysporum*, 82.2% identity with the myosin1 (A6SED8.2) motor domain from *B. cinerea*, 87.4% identity with the myosin1 (A4RE77.1) motor domain from *M. oryzae*, and 70.1% identity with the myosin (CCU80136.1) motor domain from *B. graminis* (see Supplementary Fig. S2 online). The results indicated that the reduced activity of phenamacril among these pathogens, as reported by Li *et* *al*. (2008)^10^, is was associated with a lower identity value for the myosin-5 motor domain.

### Colony morphology and hyphal growth of myosin-5 mutants

Compared with wild-type phenamacril-sensitive strain 2021, the *F. graminearum* reference strain PH-1 and the corresponding resistant strain YP-1 produced slightly smaller colonies (Fig. 3b) and had a slightly reduced hyphal growth rate (Table 3) on PDA. However, resistant strains Y2021B and Y2021F had distinctive colony morphologies (Fig. 3b) and significantly reduced hyphal growth rates (Table 3). In addition, Y2021B had white aerial hyphae. On the other hand, the colony morphology of the transformed mutants did not significantly change (Fig. 3b), which indicated that the mutations of myosin-5 did not cause the change in colony phenotype.

### RNA-Seq mapping statistics and transcriptomic analysis

We compared transcripts for the *F. graminearum* wild-type sensitive strain 2021 and the drug-induced resistant strain Y2021A. Of the total reads, approximately 84% of the total reads could be mapped to the PH-1 reference genome. The uniquely mapped reads were 80% for 2021 and 79% for Y2021A. Of the total reads, 16% had no match to the genome for 2021 or for the genome of Y2021A (Table 4). Compared with strain 2021, there are overall 10,987 expressed genes in resistant strain Y2021A, of which 350 are upregulated and 410 are downregulated by at least 2-fold. Gene expression levels were measured through short reads mapping in RPKM^36^ adjusted by a normalization factor^37^.

Based on the analysis of Gene Ontology (GO) categories, these 760 differentially expressed genes (FDR<0.01, fold-change>2) were categorized into three major functional groups: cellular component, molecular function, and biological process. The abundant genes (representing >10% of the expressed genes) were categorized into eight major functional groups based on the GO categories after exclusion of crystallin, rRNA, and mitochondrial genes. The top four functional categories included metabolic process, cellular process, binding, and catalytic activity (see Supplementary Fig. S4 online). Compared to the 2021 strain transcriptome, the 760 differentially expressed genes of strain Y2021A were involved in 171 pathways, in which the majority of the genes were involved in microbial metabolism in diverse environments, ribosome biogenesis in eukaryotes, and several molecular metabolism or degradation (see Supplementary Table S5 online).

To validate the RNA-seq results in this study, 10 genes were selected for real-time quantitative RT-PCR (qRT-PCR) analysis in parallel with the RNA-Seq analysis. The fold-changes in gene expression as determined by RNA-Seq analysis were correlated with transcript levels measured using qRT-PCR analysis (see Supplementary Fig. S3 online), which indicated that the RNA-Seq provided reliable estimates of transcript levels.

### qRT-PCR analysis of the expression of myosin-5, myosin-2B, and type II myosin genes in *F. graminearum*

**Figure 4:**
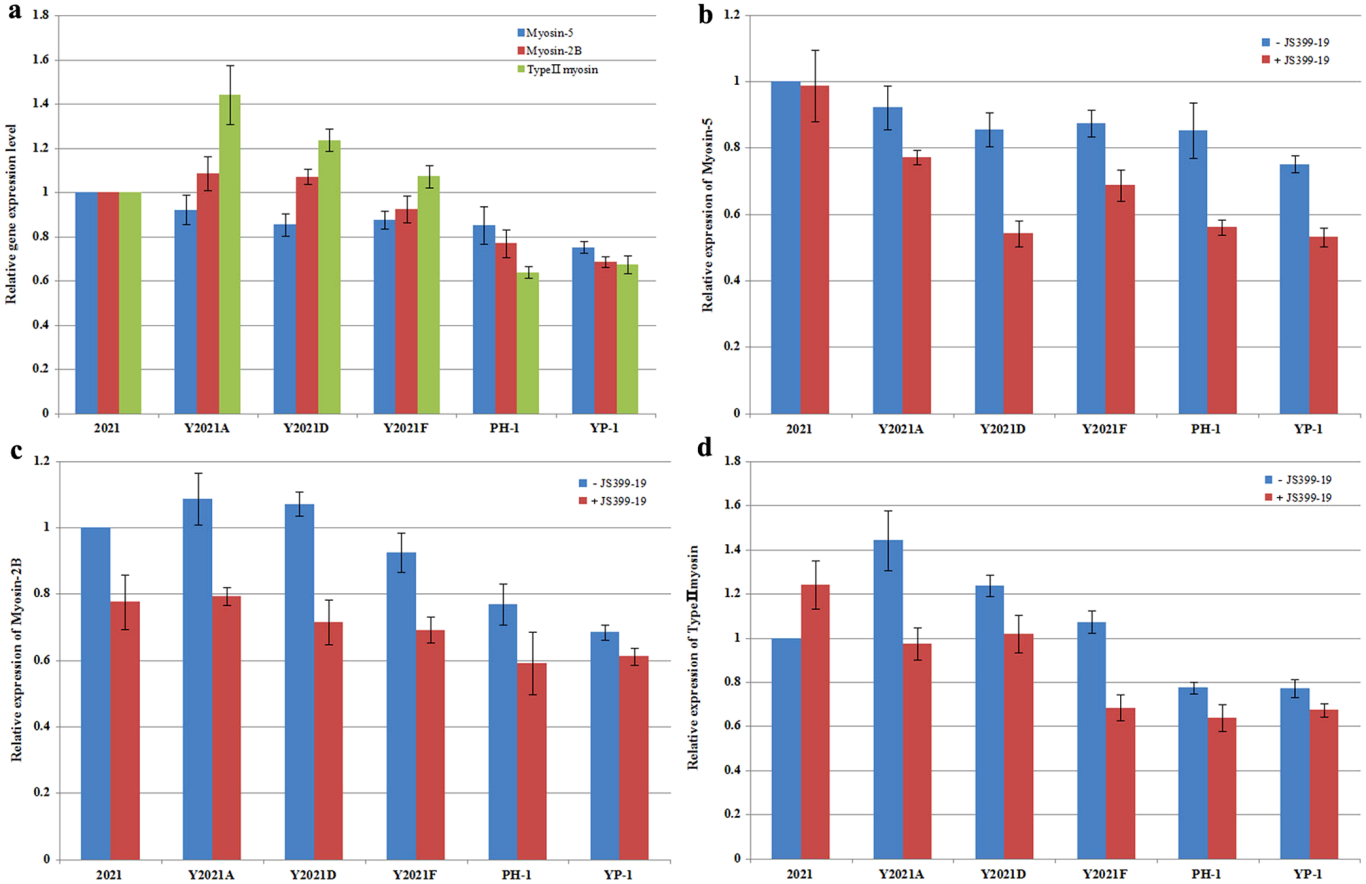
Expression level of myosin-5, myosin-2B and type II myosin in PH-1 and resistant mutants relative to that in 2021. In (b), (c) and (d), -JS399-19 represents strains treated without JS399-19 and + JS399-19 represents strains treated with JS399-19 at 0.56 μg/ml for 6h. Values are the means ± standard error (SE) of three repeated experiments.

*F. graminearum* contains three members of the myosin gene family, including the type II myosin gene FGSG_08719.1, myosin-2B (FGSG_07469.1), and myosin-5 (FGSG_01410.1). According to the RNA-seq results, the expression of the three genes did not significantly differ in the phenamacril-resistant strain Y2021A vs. the sensitive strain 2021. Furthermore, after strain 2021 and Y2021A were treated with JS399-19 at 0.21 μg/ml and 204 μg/ml (a concentration of the EC_50_ value against 2021 and Y2021A) for 6h respectively, the expression of the three genes were slightly up-regulated compared with strain 2021 and Y2021A, respectively (see Supplementary Table S6 online). To further investigate whether phenamacril resistance is related to the expression of myosin genes in *F. graminearum*, we analysed the expression levels of the three myosin genes by qRT-PCR using RNA samples of the sensitive strains 2021 and PH-1 and of the resistant strains YP-1, Y2021A, D, and F. Compared with strain 2021, the expression levels of the three myosin genes did not significantly differ among these strains (Fig. 4a). After these strains were treated with JS399-19 at 0.56 μg/ml (a concentration of the EC_90_ value against 2021) for 6h, the expression levels of the three myosin genes in resistant strains were slightly down-regulated (Fig. 4b, c and d). These results indicate that phenamacril resistance in *F. graminearum* can not be explained simply by the expression of the myosin-5 gene.

## Discussion

In this study, we used whole-genome sequencing and other molecular approaches to increase our understanding of phenamacril resistance and mode of action in *F. graminearum*. This is the first reported research to use whole-genome sequencing to investigate fungal responses to phenamacril. Phenamacril is a *Fusarium*-specific cyanoacrylate fungicide used for control of FHB. Researchers have established that cyanoacrylates inhibit photosystem II (PS II) electron transport at a common binding domain on the D1 protein of the PS II reaction center in weeds^38^,^39^,^40^. However, the mechanisms of phenamacril resistance in *Fusarium* spp. were unknown before the current study. In this research, we determined that mutations in myosin-5 confer resistance to phenamacril in *F. graminearum*.

In this study, genome-wide sequencing yielded a broad set of candidate genes. A total of 132 genes were found to have non-synonymous amino acid mutations in the resistant strain YP-1 relative to the *F. graminearum* reference strain PH-1. Because more than half of the mutated genes lack functional annotation (see Supplementary Table S2 online), however, extrapolations were limited. To confirm whether these non-synonymous mutations are related to phenamacril resistance, we sequenced 22 functional annotated genes of another *F. graminearum* sensitive strain (strain 2021) and its corresponding resistant strains. Finally, we found that all resistant mutants had point mutations in myosin-5. Furthermore, in total of 1,989 SNPs, which there are 1,208 homozygous SNPs, were mainly A to G and T to C transition mutations. This fungicide induced mutational bias shorten the time of resistant strains' occurrence in the fields.

In the three groups of cytoskeletal motors, only myosins move along tracks composed of actin filaments; kinesins and dyneins move along microtubule tracks^26^. The disorder of either the actin cytoskeleton or the microtubule cytoskeleton could affect mycelial growth and even lead to death in *F. graminearum*. In the three myosin genes of *F. graminearum*, myosin-5 is homologous to yeast Myo3p and Myo5p. Deletion of Myo3p or Myo5p in yeast results in no observable phenotypic consequences, but deletion of both is lethal^27^. This indicated that we had to use gene replacement rather than gene deletion to investigate the relationship between myosin-5 and phenamacril resistance.

The myosin-5 protein has three domains: the head or motor domain, which contains binding sites for ATP and actin; the tail homology domain 1 (TH1), and the SH3 domain. Among phenamacril-resistant mutants of *F. graminearum*, four amino acid mutations (at codon 216, 217, 418 and 420) were detected in the motor domain, and only one mutation (at codon 786) was detected in the TH1 domain. Because mutants Y2021F and YP-1 have the same mutation at codon 217, which does not result in a significant change in EC_50_ value, we inferred that the mutation at codon 786 is not central to phenamacril resistance and may not be involved at all in phenamacril resistance. The alignment of the amino acid sequences of the myosin-5 motor domain with those from *F. oxysporum*, *B. cinerea*, *M. oryzae*, and *B. graminis* indicated that the five myosin motor domains have a conserved ATP-binding site, SH1 helix, and even codon 216, 217, 418 and 420, except that *B. cinerea* myosin1 motor at codon 217 and *B. graminis* myosin motor at 418 (see Supplementary Fig. S2 online). However, phenamacril demonstrated little or no activity against mycelial growth of *B. cinerea*, *M. oryzae*, or *B. graminis*^10^. These results indicated that the different homology of the five myosin motors may lead to the change of myosin motor protein conformation which phenamacril was hard to bind.

Among the resistant mutants in this study, strain Y2021B and Y2021F had a distinctive colony morphology and a significantly reduced rate of hyphal growth. However, when the sensitive strain 2021 was transformed with the resistant fragments of Y2021B and Y2021F, the colony morphologies of 2021-22^B-myo5^ and 2021-13^F-myo5^ were not restored to the phenotypes of Y2021B and Y2021F. In addition to having mutations that increased resistance, Y2021B and Y2021F may have had other mutations that affected colony morphology and growth rate. However, the mutations of myosin-5 only increased phenamacril resistance and did not affect morphology.

The transcription of genes encoding components of a multi-enzyme pathway is often regulated in response to the availability of cofactors, precursors, intermediates, or products of the same pathway. Drugs that selectively inhibit a pathway enzyme and thereby cause an accumulation of precursors and a depletion of products can be expected to selectively induce changes in the transcription of genes coding for enzymes that comprise the affected pathway^42^. Myosin-5 is an actin-dependent ATPase motor in *F. graminearum*, and the mutations of myosin-5 might lead to the change in the expression of itself or of other corresponding genes in the resistant strain. However, the fungicide-induced resistant strain Y2021A might have other mutations in its genome, which increases the difficulty of analysis. The differentially expressed genes in strain Y2021A relative to stain 2021 are mainly distributed in metabolic process, binding, and catalytic activity and are involved in the pathway of molecular metabolism or degradation. However, these differentially expressed genes in resistant strain Y2021A can not directly reflect the mutations of myosin-5 on transcriptional effects.

RNA-seq and qRT-PCR analysis revealed that the expression of myosin-5, myosin-2B, and type II myosin genes is not significantly different in phenamacril- resistant vs. phenamacril-sensitive strains of *F. graminearum*. Therefore, resistance to phenamacril cannot be explained by changes in the expression of these genes. A better explanation is suggested by the homology modelling of *F. graminearum* myosin-5 motor domain, which revealed that the four mutations are located within 10 A° of each other and form a similar ‘pocket’ (Fig. S5A). In addition, the four kinds of mutations result in amino acid residues that are unlikely to form hydrogen bonds with phenamacril. Therefore, we hypothesize that phenamacril, when interacting with sensitive strains, binds with the *F. graminearum* myosin-5 motor domain. This binding interferes with myosin-5 motor activities and also affects actin assembly^43^, which leads to death or growth defects in *F. graminearum*. In resistant strains, however, mutations in myosin-5 motor domain reduce or block the phenamacril binding such that the myosin-5 motor domain functions normally.

In summary, our whole-genome sequencing revealed that mutations in *F. graminearum* myosin-5 confer resistance of the fungus to the fungicide phenamacril. The results also suggest that phenamacril's mode of action involves binding to the myosin-5 motor domain and subsequent effects on motor activity and actin assembly. Future research should examine the combination of the myosin-5 motor domain with phenamacril *in vitro* and the co-crystallization of myosin-5 motor domain and phenamacril. Finally, the information obtained in this study could be useful for the development of new fungicides or other drugs.

## Methods

### Strains and culture conditions

The strains used in this study are listed in Table S4 and included the *F. graminearum* reference strain PH-1^41^. The phenamacril-resistant strain YP-1, generated from PH-1 by phenamacril treatment, was subjected to whole-genome sequencing. The wild-type sensitive strain 2021 and phenamacril-induced resistant strain Y2021A were used as recipient strains for transformation. For phenotypic and mycelial growth rate analysis, all strains were grown at 25°C on PDA (200 g of potato, 20 g of glucose, 15 g of agar and 1 L of water) for 3 days. The experiment was performed three times. Growth rate among strains was compared by analysis of variance (ANOVA) and Fisher's protected least significant difference (LSD) using Data Processing System (DPS) software (Hangzhou Reifeng Information Technology Ltd., Hangzhou, China).

### Genome sequencing

Genome sequencing was performed by Shanghai Hanyu Bio-tech. Briefly, the genomic DNA of YP-1 was extracted with a Plant Genomic DNA kit (Tiangen). A 300-bp paired-end library was constructed for a 5-μg sample of YP-1 genomic DNA following the standard Illumina paired-end protocol with a 10-cycle PCR; sequencing was performed on the Illumina Hiseq 2500 with 300 cycles after cluster generation. After low quality reads were filtered, the remaining reads were mapped to the PH-1 reference genomic sequence using bowtie and bwa, which identified all of the SNPs and short indels.

### Cloning and sequence analysis of 22 functionally annotated genes

Genomic DNA of the phenamacril-sensitive strain 2021 of *F. graminearum* and of the resistant strains Y2021A, B, C, D, and F was extracted by the conventional phenol-chloroform extraction method. A total of 22 functionally annotated genes were amplified from the genomic DNA of these six strains with the primers listed in Table S1. The resultant PCR products were sequenced directly, and the results were aligned with Bioedit software.

### Construction of replacement vectors for myosin-5 using double-joint PCR and transformation

The 3-kb wild-type myosin-5 gene fragment of *F. graminearum* was amplified from strain 2021 with primers A3/A4 (Table S1). The HPH resistance gene containing the *Aspergillus nidulans* trpC promoter was amplified from plasmid pKHt^44^ with primers hphF/hphR (Table S1). A 1.2-kb upstream flanking fragment of myosin-5, which is located 168 bp upstream from the start codon, was amplified from strain 2021 and from the other resistant strains with primers A1/A2 (Table S1). In resistant strains, the 3-kb mutated zone was amplified from the five resistant strains listed in Table 2 with primers A3/A4 (Table S1). Then, the three amplicons (upstream junction, trpC + HPH, and right junction) were mixed at a 1:3:1 molar ratio and used as a template for the fusion round without primers. Finally, 1 μL of product from the fusion round was used as DNA template to amplify a 6-kb DNA fragment using primers A1/A4. The constructs were used to transform protoplasts of the wild-type strain 2021 or resistant strain Y2021A. The protoplast preparation and transformation of *F. graminearum* were performed as previously described^15^. Transformants were selected on a medium containing 100 μg/mL of hygromycin B.

### Validation of myosin-5 replacements

The insertion of the myosin-5 gene and the presence of the selective marker in the locus were verified by PCR with the primers A5/A6 and A7/A8. Transformants in which the construct had been integrated into the locus yielded a 1935-bp band at the left junction and a 3726-bp band at the right junction, whereas those in which integration was ‘random’ yielded no band. The presence of each myosin-5 mutant with its particular mutation was confirmed by sequencing the mutated zone from genomic DNA extracted from the transformants using the primers A3/A4 (Table S1). We confirmed the single-copy insertion of the cassette by Southern blotting genomic DNA that was digested with SacI. A PCR fragment obtained with the primers myo5-probeF/myo5-probeR was used as a probe (Table S1). This probe distinguished between the 7164-bp band in the transformants and the 5400-bp band in the parental strain.

### Fungicide susceptibility testing

Susceptibility to the novel cyanoacrylate fungicide phenamacril (JS399-19, Fig. S5B), which was kindly provided by the Institute for the Control of Agrochemicals, Ministry of Agriculture (ICAMA), Hangzhou, China, was assessed for all strains listed in Table 2. For sensitivity assays, a mycelial plug (5 mm in diameter) taken from the margin of a 3-day-old colony was placed on the centre of a PDA plate amended with JS399-19 at 0, 0.025, 0.05, 0.1, 0.2, or 0.4 μg/mL, or at 25, 50, 100, 200, or 400 μg/mL. Three replicates for each concentration were used for each strain. After cultures were kept at 25°C for 3 days, colony diameters were measured in two perpendicular directions; the diameter (5 mm) of the original mycelial plug was subtracted from each measurement. The average of the colony diameters was used to calculate the fungicide concentration that resulted in 50% mycelial growth inhibition (observed EC_50_). The observed EC_50_ values were calculated with DPS software. The experiment was performed twice. For mycelial growth assays, JS399-19 was added to autoclaved PDA to obtain concentrations of 0, 0.25, 1, 50, and 200 μg/mL. After the cultures were kept at 25°C for 2 days, the colonies were photographed, and their morphologies were noted.

### RNA-seq and transcriptomic data analysis

Total RNA isolation as well as construction and sequencing of cDNA libraries of the *F. graminearum* strains were conducted by BGI (China). Poly(A) mRNA was isolated using oligo dT beads. Then, single-end and paired-end RNA-seq libraries were prepared following Illumina's protocols and were sequenced on the Illumina Hiseq 2000. The sequences were aligned to the *F. graminearum* reference genome PH-1 using Bowtie2^45^. Fold-changes with FDR<0.01 were considered to be statistically significant. RPKM values (reads per kb per million reads) were provided to enable comparison of relative transcript abundance among different samples. KEGG was used to identify metabolic pathways and to calculate the statistical significance of each pathway. Gene Ontology (GO) terms were assigned by WEGO^46^ through a search of the non-redundant (NR) database in NCBI.

### Quantitative RT-PCR

RNA samples were isolated with the RNAsimple kit (Tiangen) from germ tubes grown for 24 h in YEPD liquid medium (2% glucose, 0.3% yeast extract and 1% peptone). First-strand cDNA was synthesized with the PrimeScript® RT reagent kit (TaKaRa). All quantitative RT-PCRs were performed with an ABI 7500 real-time detection system (Applied Biosystems, Foster City, CA, USA). The primers used for quantitative RT-PCR analysis are listed in Table S1. The expression of the measured genes in each sample was normalized to glyceraldehyde-3-phosphate dehydrogenase (GAPDH) gene expression, and relative changes in gene expression levels were analysed with ABI 7500 SDS software (Applied Biosystems), which automatically set the baseline. Data from three biological replicates were used to calculate the mean and standard deviation.

### Homology modelling of the *F. graminearum* myosin-5 motor domain

Homology modelling was performed on the Discovery Studio 3.5 by using the crystal structure of the actin-tropomyosin-myosin complex in *Dictyostelium discoideum* (PDB ID: 4A7F, Fig. S5D); the sequence alignment between the myosin-5 motor domain and 4A7F showed 46% identity. A three-dimensional model of the *F. graminearum* myosin-5 motor domain was built. The resulting structure was viewed with the Cn3D 4.3.1 3-D structure viewer. The model of phenamacril was plotted using SYBYL-X 2.0 (Fig. S5C).

## Supplementary Material

Supplementary Information supplementary information

## Figures and Tables

**Table 1:** Statistics for whole-genome sequencing of the phenamacril-resistant strain YP-1 of *Fusarium graminearum*. Strain YP-1 was compared with the phenamacril-sensitive strain PH-1

Sample	YP-1
Total Reads (Pair)	13,980,930
Clean data (Pair)	13,646,870
Total mapped reads	25,329,227
Sequencing depth (X)	115
Genome coverage (%)	99.35
Total SNPs	1,989
Homozygous SNPs	1,208
SNPs in CDS	364
Affected genes	161
Synonymous SNPs	102
Non-synonymous SNPs	262
Genes with amino acid changes	132

**Table 2:** Nucleotide changes in the myosin-5 gene (at codons 216, 217, 418, 420, and 786) in *Fusarium graminearum* wild-type 2021 and mutants

Strain or mutant	Codon 216	Codon 217	Codon 418	Codon 420	Codon 786
PH-1	AAA (Lys)	TCA (Ser)	AGT (Ser)	GAA (Glu)	ATG (Met)
YP-1	AAA (Lys)	TA (Leu)	AGT (Ser)	GAA (Glu)	ATG (Met)
2021	AAG (Lys)	TCA (Ser)	AGT (Ser)	GAA (Glu)	ATG (Met)
Y2021A	AAG (Lys)	CA (Pro)	AGT (Ser)	GAA (Glu)	ATG (Met)
Y2021B	AG (Glu)	TCA (Ser)	AGT (Ser)	GAA (Glu)	ATG (Met)
Y2021C	AAG (Lys)	TCA (Ser)	AGT (Ser)	GA (Gly)	TG (Val)
Y2021D	AAG (Lys)	TCA (Ser)	AG (Arg)	GAA (Glu)	TG (Val)
Y2021F	AAG (Lys)	TA (Leu)	AGT (Ser)	GAA (Glu)	TG (Val)
Y2021A-10^2-myo5^	AAG (Lys)	TCA (Ser)	AGT (Ser)	GAA (Glu)	ATG (Met)
Y2021A-24^F-myo5^	AAG (Lys)	TA (Leu)	AGT (Ser)	GAA (Glu)	TG (Val)
2021-12^A-myo5^	AAG (Lys)	CA (Pro)	AGT (Ser)	GAA (Glu)	ATG (Met)
2021-22^B-myo5^	AG (Glu)	TCA (Ser)	AGT (Ser)	GAA (Glu)	ATG (Met)
2021-9^C-myo5^	AAG (Lys)	TCA (Ser)	AGT (Ser)	GA (Gly)	TG (Val)
2021-19^D-myo5^	AAG (Lys)	TCA (Ser)	AG (Arg)	GAA (Glu)	TG (Val)
2021-13^F-myo5^	AAG (Lys)	TA (Leu)	AGT (Ser)	GAA (Glu)	TG (Val)

The amino acid corresponding to each codon is presented in parentheses, and nucleotides in boxes indicate the point mutation in the given codon.

**Table 3:** Hyphal growth rate and sensitivity of the wild-type strain 2021, reference strain PH-1, and mutants of *Fusarium graminearum* to phenamacril (JS399-19)

Strain or mutant	Growth rate (cm/day)^a^	EC_50_ (μg/ml)^b^
2021	2.23 ± 0.02D	0.21
PH-1	2.07 ± 0.03E	0.26
Y2021A-10^2-myo5^	2.32 ± 0.04ABC	0.27
Y2021A-24^F-myo5^	2.33 ± 0.01AB	158
Y2021A	2.32 ± 0.03AB	204
YP-1	2.06 ± 0.03E	146
Y2021B	1.39 ± 0.03G	202
Y2021C	2.30 ± 0.05ABC	213
Y2021D	2.28 ± 0.02BCD	42
Y2021F	1.69 ± 0.03F	149
2021-12^A-myo5^	2.35 ± 0.02A	183
2021-22^B-myo5^	2.35 ± 0.02A	186
2021-9^C-myo5^	2.28 ± 0.03BCD	194
2021-19^D-myo5^	2.26 ± 0.03CD	65
2021-13^F-myo5^	2.28 ± 0.02BCD	151

^a^Growth rate was measured after incubation on PDA at 25°C for 3 days. Values are means and standard deviations of three replicates. Means in a column followed by the same letter are not significantly different (P < 0.01).

^b^EC_50_ is the fungicide concentration resulting in 50% mycelial growth inhibition. Values are means of three experiments (differences among the experiments were not significant).

**Table 4:** Summary of RNA sequencing data for a phenamacril-sensitive strain (2021) and a resistant strain (Y2021A) relative to the reference genome of *F. graminearum* PH-1

	2021		Y2021A	
Sample name	Reads number	Percentage	Reads number	Percentage
Total reads	12,048,454	100.00%	12,305,723	100.00%
Total base pairs	590,374,246	100.00%	602,980,427	100.00%
Total mapped reads	10,079,877	83.66%	10,310,790	83.79%
Reads with perfect matches	6,619,064	54.94%	6,836,019	55.55%
Reads with ≤2 bp mismatches	3,460,813	28.72%	3,474,771	28.24%
Reads with unique matches	9,636,941	79.98%	9,759,194	79.31%
Reads with multiple matches	442,936	3.68%	551,596	4.48%
Total unmapped reads	1,968,577	16.34%	1,994,933	16.21%
