# Supplementary material for: Whole-genome sequencing reveals that mutations in myosin-5 confer resistance to the fungicide phenamacril in Fusarium graminearum
Source: Sci Rep. 2015 Feb 4;5:8248. doi: 10.1038/srep08248 (PMC5389027; doi:10.1038/srep08248)
Supplement: Supplementary Information [file srep08248-s1.pdf]

# Supplementary Information

## Whole-genome sequencing reveals that mutations in myosin-5 confer resistance to the fungicide phenamacril in *Fusarium graminearum*

Zhitian Zheng, Yiping Hou, Yiqiang Cai, Yu Zhang, Yanjun Li, and Mingguo Zhou

**Fig. S1.** Frequency of changes in bases in a phenamacril-resistant strain (YP-1) of *Fusarium graminearum* as indicated by whole-genome sequencing. Changes are relative to reference strain PH-1.

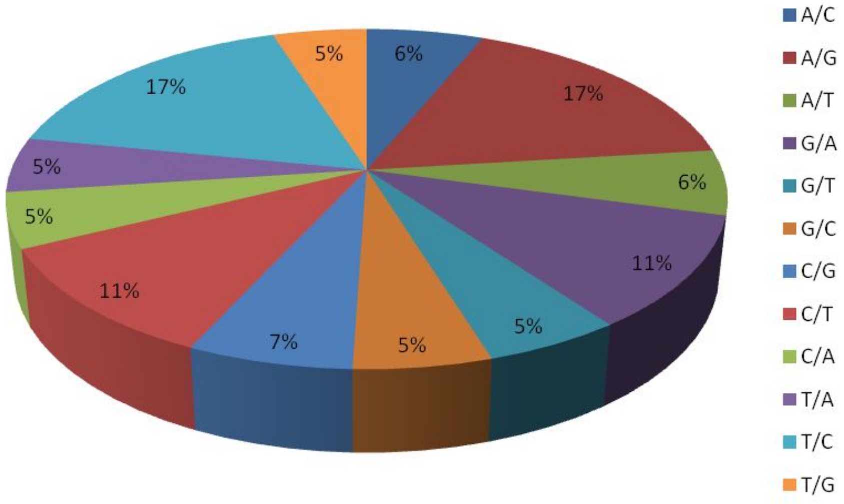


**Fig. S2.** The alignment of the amino acid sequences of myosin-5 motor domain with those from *F. oxysporum, B. cinerea, M. oryzae and B. graminis.* ATP-binding site and SH1 helix were blast by NCBI database (http://www.ncbi.nlm.nih.gov/). Codon 216 and 420 are conserved in all the five myosin motor.

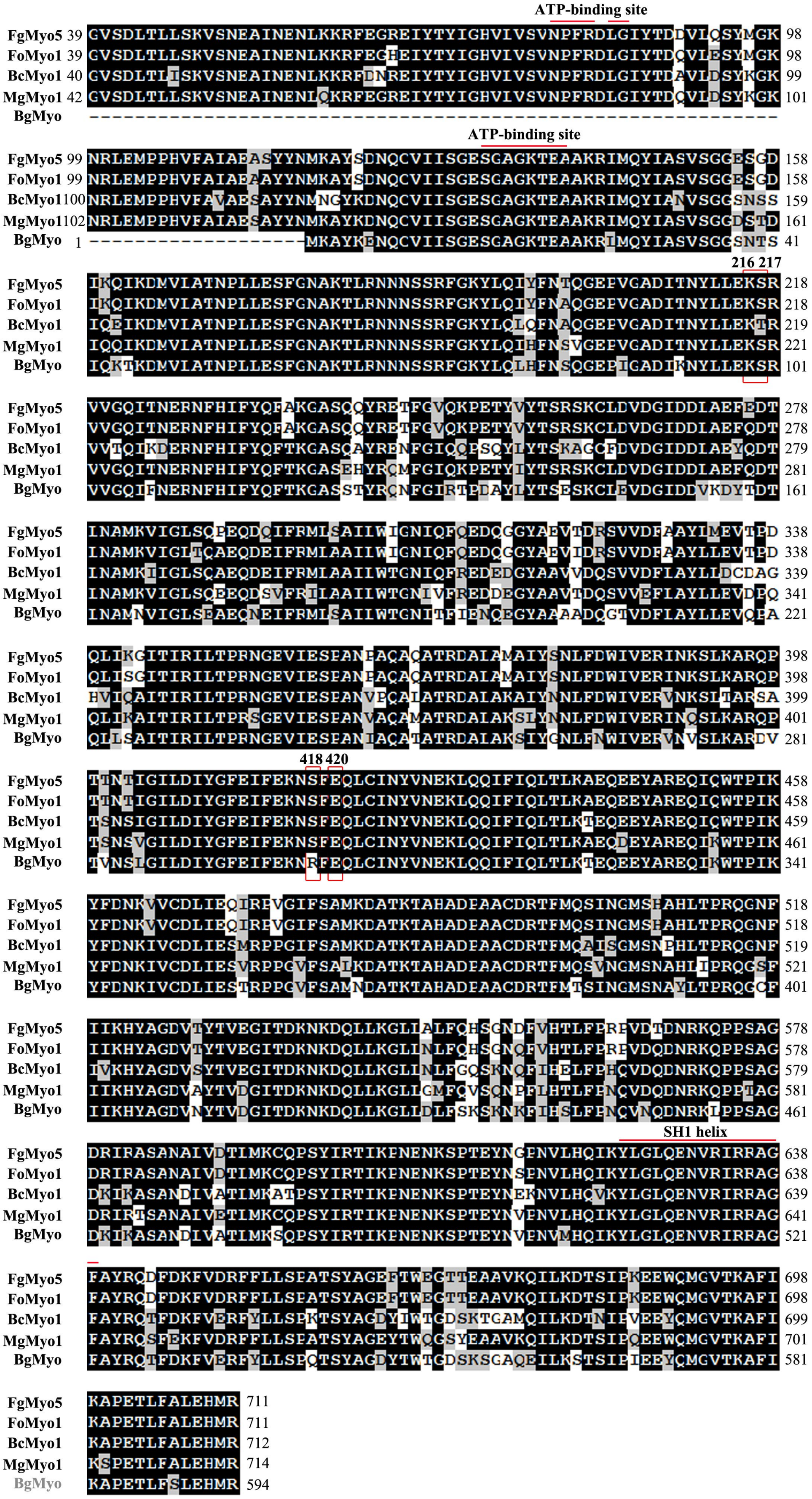
ATP-binding site
FgMyo5 39 GVSDLTLLSKVSNEAINENLKKRFEGREIYTYIGHVIVSVNPFRDLGIYTDDVLQSYMGK 98
FoMyo1 39 GVSDLTLLSKVSNEAINENLKKRFEGHEIYTYIGHVIVSVNPFRDLGIYTDQVLESYMGK 98
BcMyo1 40 GVSDLTLISKVSNEAINENLKKRFDNREIYTYIGHVIVSVNPFRDLGIYTDAVLDSYKGK 99
MgMyo1 42 GVSDLTLLSKVSNEAINENLQKRFEGREIYTYIGHVIVSVNPFRDLGIYTDQVLDSYKGK 101
BgMyo ------------------------------------------------------------
ATP-binding site
FgMyo5 99 NRLEMPPHVFAIAEASYYNMKAYSDNQCVIISGESGAGKTEAAKRIMQYIASVSGGESGD 158
FoMyo1 99 NRLEMPPHVFAIAEAAYYNMKAYSDNQCVIISGESGAGKTEAAKRIMQYIASVSGGESGD 158
BcMyo1 100 NRLEMPPHVFAVAESAYYNMNGYKDNQCVIISGESGAGKTEAAKRIMQYIANVSGGSNSS 159
MgMyo1 102 NRLEMPPHVFAIAESAYYNMKAYKDNQCVIISGESGAGKTEAAKRIMQYIASVSGGDSTD 161
BgMyo 1 -------------------MKAYKENQCVIISGESGAGKTEAAKRLMQYIASVSGGSNTS 41
216 217
FgMyo5 IKQIKDMVIATNPLLESFGNAKTLFNNNSSRFGKYLQIYFNTQGEFVGADITNYLLEKSR 218
FoMyo1 IKQIKDMVIATNPLLESFGNAKTLFNNNSSRFGKYLQIYFNAQGEFVGADITNYLLEKSR 218
BcMyo1 IQEIKDMVIATNPLLESFGNAKTLFNNNSSRFGKYLQLQFNAQGEFVGADITNYLLEKTR 219
MgMyo1 IQQIKDMVIATNPLLESFGNAKTLFNNNSSRFGKYLQIHFNSVGEFVGADITNYLLEKSR 221
BgMyo IQKTKDMVIATNPLLESFGNAKTLFNNNSSRFGKYLQLHFNSQGEPIGADIKNYLLEKSR 101
FgMyo5 VVGQITNERNFHIFYQFAKGASQQYRETFGVQKPETYVYTSRSKCLDVDGIDDIAEFEDT 278
FoMyo1 VVGQITNERNFHIFYQFAKGASQQYRETFGVQKPETYVYTSRSKCLDVDGIDDIAEFQDT 278
BcMyo1 VVTQIKDERNFHIFYQFTKGASQAYRENFGIQQPSQYLYTSKAGCFDVDGIDDIAEYQDT 279
MgMyo1 VVGQITNERNFHIFYQFTKGASEHYRQMFGIQKPETYIYTSRSKCLDVDGIDDIAEFQDT 281
BgMyo VVGQIFNERNFHIFYQFTKGASSTYRQNFGIRTPDAYLYTSESKCLEVDGIDDVKDYTDT 161
FgMyo5 INAMKVIGLSQPEQDQIFRMLSAIIWIGNIQFQEDQGGYAEVTDRSVVDFAAYIMEVTPD 338
FoMyo1 INAMKVIGLTQAEQDEIFRMIAAIIWIGNIQFQEDQGGYAEVIDRSVVDFAAYLLEVTPD 338
BcMyo1 INAMKIIGLSQAEQDEIFRMIAAIIWTGNIQFREDEDGYAAVVDQSVVDFIAYLLDCDAG 339
MgMyo1 INAMKVIGLSQEEQDSVFRILAAIIWTGNIVFREDDEGYAAVTDQSVVEFIAYLLEVDPQ 341
BgMyo INAMNVIGLSEAEQNEIFRMLSAIIWTGNITFIENQEGYAAAADQGTVDFIAYLLEVQPA 221
FgMyo5 QLIKGITIRILTPRNGEVIESPANPAQAQATRDAIAMAIYSNLFDWIVERINKSLKARQP 398
FoMyo1 QLISGITIRILTPRNGEVIESPANPAQAQATRDAIAMAIYSNLFDWIVERINKSLKARQP 398
BcMyo1 HVIQAITIRILTPRNGEVIESPANVPQALATRDAIAKAIYNNLFDWIVERVNKSITARSA 399
MgMyo1 QLIKAITIRILTPRSGEVIESPANVAQAMATRDAIAKSLYNNLFDWIVERINQSLKARQP 401
BgMyo QLLSAITIRILTPRNGEVIESPANIAQATATRDAIAKSIYGNLFNWIVERVNVSLKARDV 281
418 420
FgMyo5 TTNTIGILDIYGFEIFEKNSFEQLCINYVNEKLQQIFIQLTLKAEQEEYAREQIQWTPIK 458
FoMyo1 TTNTIGILDIYGFEIFEKNSFEQLCINYVNEKLQQIFIQLTLKAEQEEYAREQIQWTPIK 458
BcMyo1 TSNSIGILDIYGFEIFEKNSFEQLCINYVNEKLQQIFIQLTLKTEQEEYAREQIQWTPIK 459
MgMyo1 TSNSVGILDIYGFEIFEKNSFEQLCINYVNEKLQQIFIQLTLKAEQDEYAREQIKWTPIK 461
BgMyo TVNSLGILDIYGFEIFEKNRFEQLCINYVNEKLQQIFIQLTLKTEQEEYAREQIKWTPIK 341
FgMyo5 YFDNKVVCDLIEQIRPVGIFSAMKDATKTAHADPAACDRTFMQSINGMSHAHLTPRQGNF 518
FoMyo1 YFDNKVVCDLIEQIRPVGIFSAMKDATKTAHADPAACDRTFMQSINGMSHAHLTPRQGNF 518
BcMyo1 YFDNKIVCDLIESMRPPGIFSAMKDATKTAHADPAACDRTFMQAISGMSNPHLTPRQGNF 519
MgMyo1 YFDNKIVCDLIESVRPPGVFSALKDATKTAHADPAACDRTFMQSVNGMSNAHLIPRQGSF 521
BgMyo YFDNKIVCDLIESTRPPGVFSAMNDATKTAHADPAACDRTFMTSINGMSNAYLTPRQGCF 401
FgMyo5 IIKHYAGDVTYTVEGITDKNKDQLLKGLIALFQHSGNDFVHTLFPRPVDTDNRKQPPSAG 578
FoMyo1 IIKHYAGDVTYTVEGITDKNKDQLLKGLINLFQHSGNQFVHTLFPRPVDQDNRKQPPSAG 578
BcMyo1 IVKHYAGDVSYTVEGITDKNKDQLLKGLINLFGQSKNQFIHELFPHCVDQDNRKQPPSAG 579
MgMyo1 IIKHYAGDVAYTVDGITDKNKDQLLKGLLGMFQVSQNPFLHTLFPNCVDQDNRKQPPTAG 581
BgMyo IIKHYAGDVNYTVDGITDKNKDQLLKGLLDLFSKSKNKFIHSLFPNCVNQDNRKLPPSAG 461
SH1 helix
FgMyo5 DRIRASANAIVDTIMKCQPSYIRTIKPNENKSPTEYNGPNVLHQIKYLGLQENVRIRRAG 638
FoMyo1 DRIRASANAIVDTIMKCQPSYIRTIKPNENKSPTEYNSPNVLHQIKYLGLQENVRIRRAG 638
BcMyo1 DKIKASANDIVATIMKATPSYIRTIKPNENKSPTEYNEKNVLHQVKYLGLQENVRIRRAG 639
MgMyo1 DRIRTSANAIVETIMKCQPSYIRTIKPNENKSPTEYNVPNVLHQIKYLGLQENVRIRRAG 641
BgMyo DKIKASANDIVATIMKSQPSYIRTIKPNENKSPTEYNVPNVMHQIKYLGLQENVRIRRAG 521
FgMyo5 FAYRQDFDKFVDRFFLLSPATSYAGEFTWEGTTEAAVKQILKDTSIPKEEWQMGVTKAFI 698
FoMyo1 FAYRQDFDKFVDRFFLLSPATSYAGEFTWEGTTEAAVKQILKDTSIPKEEWQMGVTKAFI 698
BcMyo1 FAYRQTFDKFVERFYLLSPKTSYAGDYIWTGDSKTGAMQILKDTNIPVEEYQMGVTKAFI 699
MgMyo1 FAYRQSFEKFVDRFFLLSPATSYAGEYTWQGSYEAAVKQILKDTSIPQEEWQMGVTKAFI 701
BgMyo FAYRQTFDKFVERFYLLSPQTSYAGDYTWTGDSKSGAQEILKSTSIPIEEYQMGVTKAFI 581
FgMyo5 KAPETLFALEHMR 711
FoMyo1 KAPETLFALEHMR 711
BcMyo1 KAPETLFALEHMR 712
MgMyo1 KSPETLFALEHMR 714
BgMyo KAPETLFSLEHMR 594

**Fig. S3.** Relative expression of 10 differentially expressed genes as indicated by RNA-Seq and qRT-PCR. Values are means (± SE for qRT-PCR) of three repeated experiments.

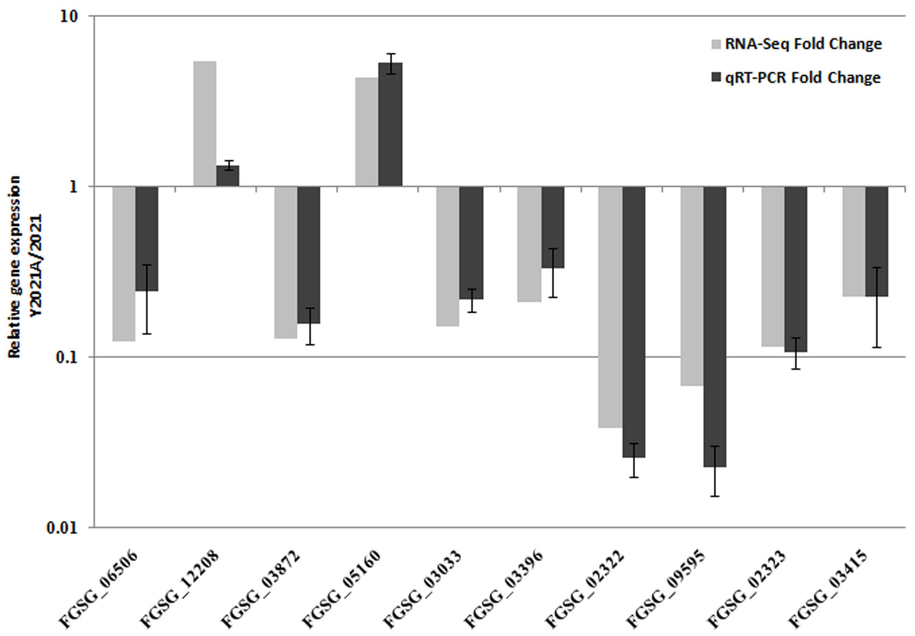


**Fig. S4.** Functional categorization of differentially expressed genes based on known genes in the Uniprot database. Gene Ontology (GO) terms at the 2nd level were plotted here, and in this ontology, “Cellular component”, “Molecular function”, and “Biological process” are categorized independently.

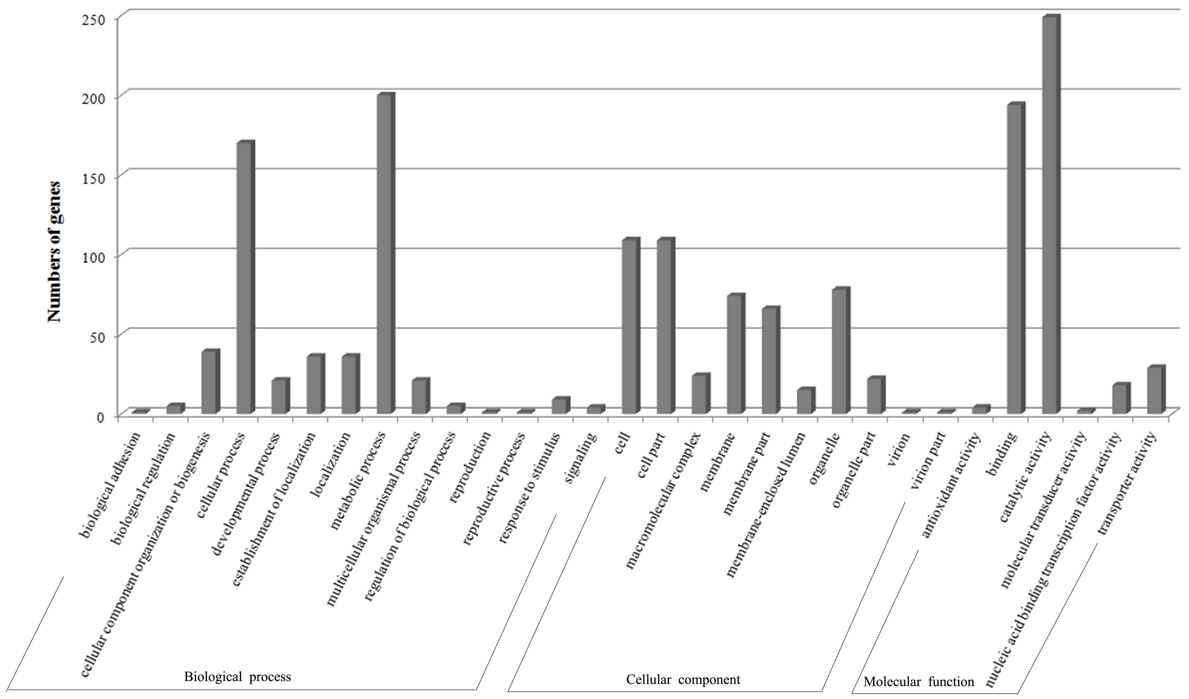


**Fig. S5.** Schematic representation of homology modelling of the *Fusarium graminearum* myosin-5 motor domain (A), general chemical structure of phenamacril (B), model of phenamacril (C), and ribbon models of myosin in *Dictyostelium discoideum* 4A7F (D). In (A) amino acids Lys216, Ser217, Ser418, and Glu420 of the myosin-5 motor domain are shown in yellow. Amino acids at these sites formed a similar ‘pocket’. In (C), blue and red represent nitrogen and oxygen atoms, respectively. In (D) Ribbon models of myosin in the crystal structure of the actin-tropomyosin-myosin complex in *Dictyostelium discoideum*, amino acids at sites 186, 187, 397 and 399 are conserved with that of the myosin-5 motor domain. These sites also formed a similar ‘pocket’.

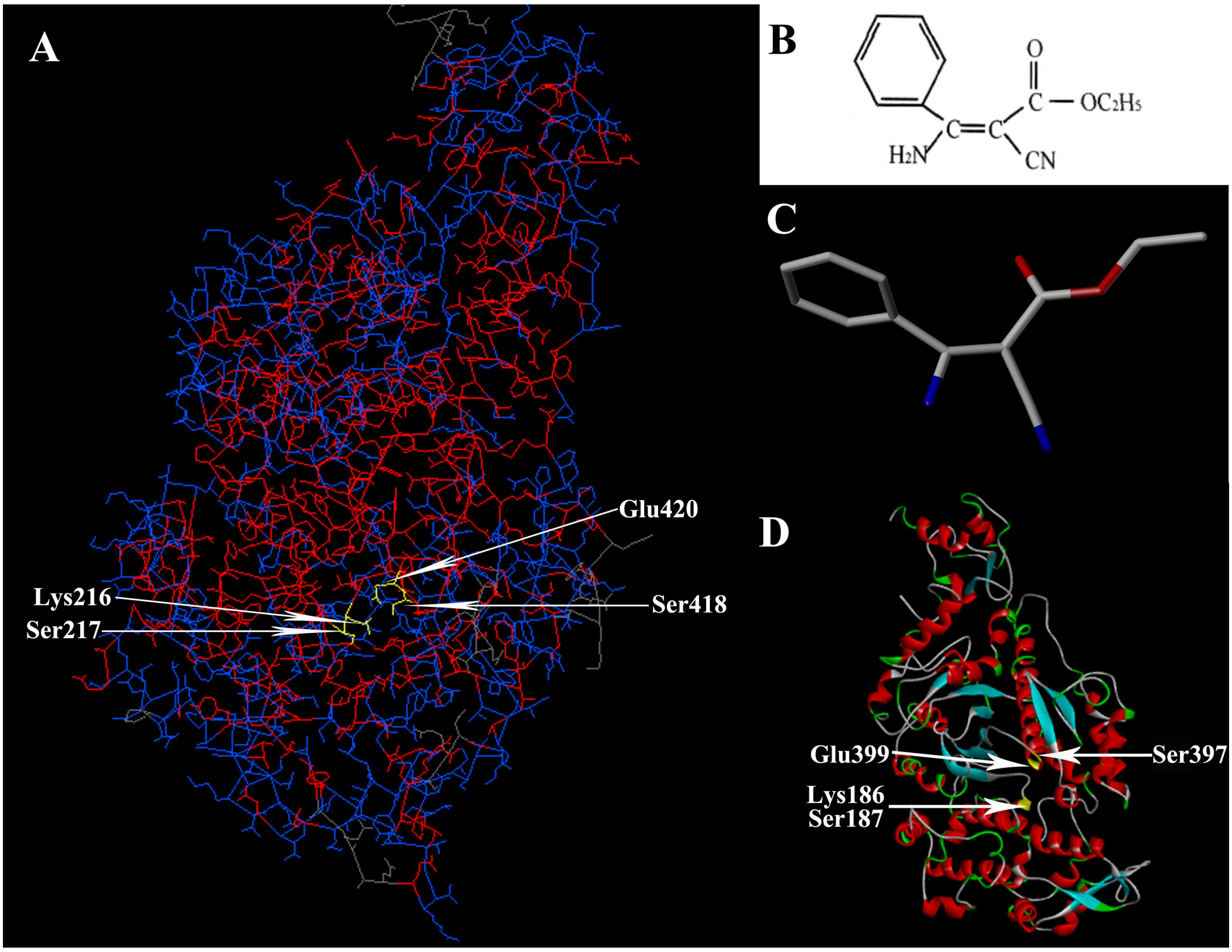


Table S1. Oligonucleotide primers used in this study.

| Primer code | Primer | Sequence (5'-3') | Relevant characteristics |
|---|---|---|---|
| 1 | A1 | TTGCGGTAGGAGAAGATG | PCR primers for amplification of the upstream *myosin-5* fragment |
| 2 | A2 | AGCCAGCCAACAGCTCCCAAGGGAAACTCTGGAAGC | |
| 3 | A3 | TACGCAAACCGCCTCTCCCCTTCCTCCGTGCCGTGATA | PCR primers for amplification of the *myosin-5* mutant zone fragment |
| 4 | A4 | TCGGCTGGGACTTGTGAA | |
| 5 | A5 | CAGGCTTGGTCTGGTTGG | A pair of PCR primers for identification of replacement integration at the left junction. |
| 6 | A6 | CCGTGGTTGGCTTGTATG | |

| | | | |
|---|---|---|---|
| 7 | A7 | ATTTGGATGCTTGGGTAG | A pair of PCR primers for identification of replacement integration at the right junction. |
| 8 | A8 | ATAGAAGCAGCGGCAGAG | |
| 9 | hphF | GGGAGCTGTTGGCTGGCTGGTGG | PCR primers for amplification of the 1.7-kb *hph* resistance gene containing the *Aspergillus nidulans* trpC promoter |
| 10 | hphR | GGGGAGAGGCGGTTTGCGTATTG | |
| 11 | myo5-probeF | GTTTACCGACATTACCGC | PCR primers to amplify the 622-bp myosin-5 upstream fragment used as probe for Southern blot |
| 12 | myo5-probeR | TTCTAGTGGTGCCTGACG | |
| 13 | Myo5-F | TCACAAGTCCCAGCCGATTA | Quantitative real-time PCR primers for analysis of *myosin-5* expression |
| 14 | Myo5-R | GGTTGCCTTGAATGCGAGA | |
| 15 | Myo2B-F | GCAGTCCCTCGGTTCTATGA | Quantitative real-time PCR primers for analysis of *myosin-2B* expression |
| 16 | Myo2B-R | CTGGTTGGCTTCCGTCTGT | |
| 17 | 08719cF | GCCTTGGACAGGGTTTCATT | Quantitative real-time PCR primers for analysis of *type II myosin* expression |
| 18 | 08719cR | TCCTCCTCAGATTCGGCAC | |
| 19 | GAPDH-F | CTTACTGCCTCCACCAACTG | Quantitative real-time PCR primers for analysis of the elongation factor 1-alpha gene expression |
| 20 | GAPDH-R | TGACGTTGGAAGGAGCGAAG | |
| 21 | 01099F | GACCGCTCGCTCAACAGA | PCR primers for amplification of the FGSG_01099 fragment |
| 22 | 01099R | CCATAATCACGCTGCTGT | |
| 23 | 01234F | CGCTCATCACTCGGCTAC | PCR primers for amplification of the FGSG_01234 fragment |
| 24 | 01234R | AGTCTGGCAACAGCATCG | |
| 25 | 01410F | TCCCGTCAGGCACCACTA | PCR primers for amplification of the FGSG_01410 fragment |
| 26 | 01410R | CGGTTGCCCAGTTCAGTC | |
| 27 | 01675F | CACTGGTTACGCTTCTCG | PCR primers for amplification of the FGSG_01675 fragment |
| 28 | 01675R | GTCAAGGCGATGAGGATA | |
| 29 | 02501F | TCCCTCCTCATCTCAACG | PCR primers for amplification of the FGSG_02501 fragment |
| 30 | 02501R | TCAAGTGTAAGGGCGGTA | |
| 31 | 03065F | CCAAGACCTGGGAACATC | PCR primers for amplification of the FGSG_03065 fragment |
| 32 | 03065R | GTACTTGGAGTGCTGGGT | |
| 33 | 03772F | ATGCCTCAGACGACAACA | PCR primers for amplification of the FGSG_03772 fragment |
| 34 | 03772R | GCAAATCTGCCTGGGACT | |
| 35 | 05615F | TCTGCTCCCGCCCACAAA | PCR primers for amplification of the FGSG_05615 fragment |
| 36 | 05615R | TGGCATCGTTGGGCATAG | |
| 37 | 05894F | GCCGAGACAATGCCACA | PCR primers for amplification of the FGSG_05894 fragment |
| 38 | 05894R | AGAGCAATGGTGGTGTCC | |
| 39 | 06998F | ACACTCGTTGGCTCTTGG | PCR primers for amplification of the FGSG_06998 fragment |
| 40 | 06998R | TGCAACCCTAACTATCCC | |
| 41 | 09397R | AGGCTTTGACAGGCTCCA | PCR primers for amplification of the FGSG_09397 fragment |
| 42 | 09397R | GCTCAGGTTCTCCTCGTC | |
| 43 | 09895F | GTCCGTGCCCTATCCTGT | PCR primers for amplification of the FGSG_09895 fragment |
| 44 | 09895R | TTCGCTCCGTCACAAACA | |
| 45 | 10358F | GCCAGTTGGTGTCATTCT | PCR primers for amplification of the FGSG_10358 fragment |
| 46 | 10358R | TTCGGAAACAAGGAATGA | |

| | | | |
|---|---|---|---|
| 47 | 11973F | CCTCGTTCCTTGGCTGTA | PCR primers for amplification of the |
| 48 | 11973R | CGACCATACGCCAGTTGA | FGSG_11973 fragment |
| 49 | 12039F | TACCTGCGTTGAGGAAGA | PCR primers for amplification of the |
| 50 | 12039R | CTTTCGCCAGCAAACTCT | FGSG_12039 fragment |
| 51 | 12129F | CTGTTGCGGTGGATGTCA | PCR primers for amplification of the |
| 52 | 12129R | CCAAACGCCAACTGACTG | FGSG_12129 fragment |
| 53 | 12369F | GTTCTCGCCACAGTTCAA | PCR primers for amplification of the |
| 54 | 12369R | TGGTAGAACGGTCGGTGG | FGSG_12369 fragment |
| 55 | 12745F | GGACCACCTTATTACCGC | PCR primers for amplification of the |
| 56 | 12745R | GCGGATGTTGTTGCGTAG | FGSG_12745 fragment |
| 57 | 13343F | GCACTGGTTCGTCGGTAG | PCR primers for amplification of the |
| 58 | 13343R | GCAGAGGTCCGAGTTAGA | FGSG_13343 fragment |
| 59 | 13620F | AGTTTCGTTCTTTCCCTC | PCR primers for amplification of the |
| 60 | 13620R | AATGGAGACAATCTACCG | FGSG_13620 fragment |
| 61 | 13823F | GATGGTGGACAAGATGAC | PCR primers for amplification of the |
| 62 | 13823R | TCCAGCCTTGAAATACTC | FGSG_13823 fragment |
| 63 | 13897F | CGCAAGGACACTACCAAC | PCR primers for amplification of the |
| 64 | 13897R | AGAGCAAGGAGCCAATCA | FGSG_13897 fragment |
| 65 | 02322rtF | TCTGGTGGTGTCATCAACGG | Quantitative real-time PCR primers for |
| 66 | 02322rtR | ATAAGACCGCCACCGACTGA | analysis of FGSG_02322 expression |
| 67 | 02323rtF | CAGCAAGCACAATACCGCAG | Quantitative real-time PCR primers for |
| 68 | 02323rtR | TGGGATGGTAAACCGAGAGC | analysis of FGSG_02323 expression |
| 69 | 03033rtF | GCAATGTGCTTCAGTTTCGG | Quantitative real-time PCR primers for |
| 70 | 03033rtR | AACAACAGCCATCGCATACC | analysis of FGSG_03033 expression |
| 71 | 03396rtF | CGTCGGTGTTATCAGTGCCA | Quantitative real-time PCR primers for |
| 72 | 03396rtR | CAAATCGTCCTGCCAATGCT | analysis of FGSG_03396 expression |
| 73 | 03415rtF | GCATCGCAAATGGCAGTG | Quantitative real-time PCR primers for |
| 74 | 03415rtR | AGCATTGCGGAGCGACATAG | analysis of FGSG_ 03415 expression |
| 75 | 03872rtF | ATCGCTGGTATCGGTTCGTG | Quantitative real-time PCR primers for |
| 76 | 03872rtR | CGGAGTGCTGAGGATGAATG | analysis of FGSG_03872 expression |
| 77 | 05160rtF | CTTTGCTGGCTGCTGTGTTG | Quantitative real-time PCR primers for |
| 78 | 05160rtR | ATAGCGTTGAAGATGCCCCA | analysis of FGSG_05160 expression |
| 79 | 06506rtF | TGGTTACGCCAATGTTTCCG | Quantitative real-time PCR primers for |
| 80 | 06506rtR | CAGCGTTGGCAGCATAGTTC | analysis of FGSG_06506 expression |
| 81 | 09595rtF | GCAACTGGCGACTCATTCTG | Quantitative real-time PCR primers for |
| 82 | 09595rtR | TGAGCACGAGGGGAATGGT | analysis of FGSG_09595 expression |
| 83 | 12208rtF | GGACACTTCCCGACAACACA | Quantitative real-time PCR primers for |
| 84 | 12208rtR | AAGCCCACGATTTCTTGTTG | analysis of FGSG_12208 expression |

Table S2. Statistics of 132 genes with amino acid mutations.

| Gene | Description | Synonymous SNPs | Non-synonymous SNPs |
|---|---|---|---|
| FGSG_00100 | hypothetical protein | 1 | 0 |
| FGSG_00180 | hypothetical protein | 1 | 3 |
| FGSG_00410 | hypothetical protein | 0 | 1 |
| FGSG_00554 | hypothetical protein | 0 | 1 |
| FGSG_00614 | hypothetical protein | 2 | 2 |
| FGSG_00727 | hypothetical protein | 2 | 11 |
| FGSG_00924 | hypothetical protein | 0 | 1 |
| FGSG_00925 | hypothetical protein | 0 | 2 |
| FGSG_01099 | GTP-binding nuclear protein GSP1/Ran | 0 | 2 |
| FGSG_01116 | hypothetical protein | 0 | 1 |
| FGSG_01156 | hypothetical protein | 0 | 1 |
| FGSG_01163 | hypothetical protein | 2 | 5 |
| FGSG_01234 | adenylate cyclase | 0 | 2 |
| FGSG_01410 | myosin-5 | 0 | 1 |
| FGSG_01434 | hypothetical protein | 0 | 2 |
| FGSG_01501 | hypothetical protein | 1 | 2 |
| FGSG_01545 | hypothetical protein | 0 | 1 |
| FGSG_01641 | hypothetical protein | 0 | 1 |
| FGSG_01675 | dicarboxylic amino acid permease | 0 | 2 |
| FGSG_01899 | hypothetical protein | 0 | 1 |
| FGSG_01927 | hypothetical protein | 0 | 1 |
| FGSG_01981 | hypothetical protein | 0 | 2 |
| FGSG_02229 | hypothetical protein | 1 | 2 |
| FGSG_02501 | transcriptional repressor rco-1 | 1 | 3 |
| FGSG_02594 | hypothetical protein | 0 | 1 |
| FGSG_02679 | hypothetical protein | 0 | 1 |
| FGSG_02799 | hypothetical protein | 0 | 1 |
| FGSG_02896 | hypothetical protein | 0 | 1 |
| FGSG_03065 | phytoene dehydrogenase | 0 | 6 |
| FGSG_03191 | hypothetical protein | 0 | 2 |
| FGSG_03550 | hypothetical protein | 2 | 4 |
| FGSG_03772 | drug resistance protein | 0 | 1 |
| FGSG_04091 | hypothetical protein | 0 | 1 |
| FGSG_04189 | hypothetical protein | 3 | 4 |
| FGSG_04370 | hypothetical protein | 0 | 2 |
| FGSG_04373 | hypothetical protein | 0 | 1 |
| FGSG_04439 | hypothetical protein | 0 | 2 |
| FGSG_04871 | hypothetical protein | 1 | 2 |
| FGSG_04890 | hypothetical protein | 0 | 3 |
| FGSG_04987 | hypothetical protein | 0 | 2 |
| FGSG_05347 | hypothetical protein | 5 | 5 |

| | | | |
|---|---|---|---|
| FGSG_05615 | adenosylhomocysteinase | 0 | 1 |
| FGSG_05794 | hypothetical protein | 2 | 4 |
| FGSG_05883 | hypothetical protein | 0 | 2 |
| FGSG_05894 | serine/threonine protein phosphatase 2A | 1 | 2 |
| FGSG_06175 | hypothetical protein | 0 | 1 |
| FGSG_06302 | hypothetical protein | 0 | 1 |
| FGSG_06431 | hypothetical protein | 0 | 2 |
| FGSG_06445 | hypothetical protein | 0 | 1 |
| FGSG_06998 | glucan synthesis regulatory protein | 4 | 12 |
| FGSG_07013 | hypothetical protein | 0 | 1 |
| FGSG_07078 | hypothetical protein | 0 | 1 |
| FGSG_07133 | hypothetical protein | 0 | 1 |
| FGSG_07525 | hypothetical protein | 2 | 5 |
| FGSG_07526 | hypothetical protein | 0 | 1 |
| FGSG_07562 | hypothetical protein | 0 | 2 |
| FGSG_07807 | hypothetical protein | 0 | 1 |
| FGSG_08249 | hypothetical protein | 0 | 1 |
| FGSG_08608 | hypothetical protein | 0 | 1 |
| FGSG_08701 | hypothetical protein | 0 | 1 |
| FGSG_08759 | hypothetical protein | 0 | 1 |
| FGSG_08842 | hypothetical protein | 0 | 5 |
| FGSG_08955 | hypothetical protein | 2 | 3 |
| FGSG_09281 | hypothetical protein | 2 | 4 |
| FGSG_09318 | hypothetical protein | 0 | 3 |
| FGSG_09329 | hypothetical protein | 0 | 2 |
| FGSG_09397 | glycylpeptide N-tetradecanoyltransferase | 0 | 2 |
| FGSG_09478 | hypothetical protein | 1 | 1 |
| FGSG_09605 | hypothetical protein | 0 | 2 |
| FGSG_09686 | hypothetical protein | 2 | 1 |
| FGSG_09762 | hypothetical protein | 2 | 1 |
| FGSG_09876 | hypothetical protein | 0 | 1 |
| FGSG_09889 | hypothetical protein | 0 | 2 |
| FGSG_09895 | neutral trehalase | 0 | 1 |
| FGSG_09950 | hypothetical protein | 0 | 2 |
| FGSG_10075 | hypothetical protein | 2 | 2 |
| FGSG_10333 | hypothetical protein | 1 | 1 |
| FGSG_10351 | hypothetical protein | 0 | 1 |
| FGSG_10357 | hypothetical protein | 0 | 4 |
| FGSG_10358 | GMP synthase | 5 | 3 |
| FGSG_10488 | hypothetical protein | 0 | 1 |
| FGSG_10577 | hypothetical protein | 1 | 1 |
| FGSG_10620 | hypothetical protein | 3 | 3 |

| | | | |
|---|---|---|---|
| FGSG_10807 | hypothetical protein | 0 | 2 |
| FGSG_10812 | hypothetical protein | 0 | 2 |
| FGSG_10932 | hypothetical protein | 0 | 1 |
| FGSG_11097 | hypothetical protein | 0 | 1 |
| FGSG_11615 | hypothetical protein | 1 | 2 |
| FGSG_11638 | hypothetical protein | 0 | 1 |
| FGSG_11673 | hypothetical protein | 0 | 1 |
| FGSG_11710 | hypothetical protein | 0 | 1 |
| FGSG_11862 | hypothetical protein | 1 | 2 |
| FGSG_11884 | hypothetical protein | 0 | 1 |
| FGSG_11912 | hypothetical protein | 2 | 3 |
| FGSG_11936 | hypothetical protein | 1 | 3 |
| FGSG_11973 | phosphate-repressible phosphate permease | 1 | 1 |
| FGSG_11981 | hypothetical protein | 0 | 2 |
| FGSG_12017 | hypothetical protein | 0 | 1 |
| FGSG_12039 | chitin synthase 6 | 0 | 1 |
| FGSG_12040 | hypothetical protein | 1 | 1 |
| FGSG_12086 | hypothetical protein | 1 | 1 |
| FGSG_12129 | peroxisomal copper amine oxidase | 0 | 4 |
| FGSG_12178 | hypothetical protein | 0 | 2 |
| FGSG_12270 | hypothetical protein | 0 | 2 |
| FGSG_12323 | hypothetical protein | 2 | 1 |
| FGSG_12369 | peroxidase/catalase 2 | 0 | 1 |
| FGSG_12439 | hypothetical protein | 3 | 3 |
| FGSG_12492 | hypothetical protein | 0 | 4 |
| FGSG_12595 | hypothetical protein | 0 | 2 |
| FGSG_12745 | heat shock protein HSP98 | 0 | 1 |
| FGSG_12781 | hypothetical protein | 0 | 2 |
| FGSG_12827 | hypothetical protein | 0 | 1 |
| FGSG_12946 | hypothetical protein | 0 | 1 |
| FGSG_12980 | hypothetical protein | 0 | 1 |
| FGSG_12985 | hypothetical protein | 0 | 1 |
| FGSG_13148 | hypothetical protein | 0 | 1 |
| FGSG_13181 | hypothetical protein | 3 | 1 |
| FGSG_13292 | hypothetical protein | 0 | 1 |
| FGSG_13342 | hypothetical protein | 1 | 1 |
| FGSG_13343 | glycerol kinase | 0 | 1 |
| FGSG_13561 | hypothetical protein | 0 | 2 |
| FGSG_13581 | hypothetical protein | 0 | 1 |
| FGSG_13620 | alpha-1,2 mannosyltransferase KTR1 | 0 | 1 |
| FGSG_13634 | hypothetical protein | 0 | 2 |
| FGSG_13710 | hypothetical protein | 0 | 1 |

| | | | |
|---|---|---|---|
| FGSG_13715 | hypothetical protein | 0 | 4 |
| FGSG_13823 | aconitate hydratase | 1 | 1 |
| FGSG_13897 | amino-acid permease inda1 | 0 | 1 |
| FGSG_13946 | hypothetical protein | 0 | 3 |
| FGSG_13966 | hypothetical protein | 1 | 2 |
| FGSG_13987 | hypothetical protein | 0 | 5 |
| FGSG_14031 | hypothetical protein | 0 | 3 |

Table S3. Statistics of base variable frequency in *Fusarium graminearum* sequencing resistant strain YP-1.

| Base changes | Numbers of site |
|---|---|
| A/C | 123 |
| A/G | 336 |
| A/T | 123 |
| G/A | 213 |
| G/T | 105 |
| G/C | 105 |
| C/G | 126 |
| C/T | 215 |
| C/A | 104 |
| T/A | 104 |
| T/C | 336 |
| T/G | 99 |

Table S4. *Fusarium graminearum* strains used in this study.

| Strain or mutant | Genotype | Reference |
|---|---|---|
| 2021 | Wild-type, phenamacril-sensitive strain | 47 |
| PH-1 | Reference strain | 41 |
| Y2021A-10$^{2\text{-}myo5}$ | Myosin-5 replaced by 2021's myosin-5 fragment mutant in Y2021A genetic background | This study |
| Y2021A-24$^{F\text{-}myo5}$ | Myosin-5 replaced by Y2021F's myosin-5 fragment mutant in Y2021A genetic background | This study |
| YP-1 | Phenamacril-resistant strain generated from the reference strain PH-1 by fungicide treatment | This study |
| Y2021A | Phenamacril-resistant strains generated from the wild-type strain 2021 by fungicide treatment | 47 |
| Y2021B | | This study |
| Y2021C | | This study |
| Y2021D | | This study |
| Y2021F | | This study |
| 2021-12$^{A\text{-}myo5}$ | Myosin-5 replaced by Y2021A's myosin-5 fragment mutant in 2021 genetic background | This study |
| 2021-22$^{B\text{-}myo5}$ | Myosin-5 replaced by Y2021B's myosin-5 fragment mutant in 2021 genetic background | This study |
| 2021-9$^{C\text{-}myo5}$ | Myosin-5 replaced by Y2021C's myosin-5 fragment mutant in 2021 genetic background | This study |
| 2021-19$^{D\text{-}myo5}$ | Myosin-5 replaced by Y2021D's myosin-5 fragment mutant in 2021 genetic background | This study |
| 2021-13$^{F\text{-}myo5}$ | Myosin-5 replaced by Y2021F's myosin-5 fragment mutant in 2021 genetic background | This study |

Table S5. Pathway of the more differentially expressed genes based on the KEGG database.

| Pathway | Up-regulated genes | Down-regulated genes |
|---|---|---|
| Drug metabolism cytochrome P450 | FGSG_07685 | FGSG_02291, FGSG_04214, FGSG_03915, FGSG_10200, FGSG_05382, FGSG_11097, FGSG_04194 |
| Microbial metabolism in diverse environments | FGSG_13963, FGSG_07685, FGSG_12344, FGSG_11542, FGSG_02217, FGSG_13962, FGSG_07080, FGSG_02906, FGSG_07838, FGSG_00489, FGSG_01692, FGSG_03996, | FGSG_02291, FGSG_10198, FGSG_01581, FGSG_05623, FGSG_09572, FGSG_04214, FGSG_08875, FGSG_00321, FGSG_13444, FGSG_08463, FGSG_10120, FGSG_08443, FGSG_01433, FGSG_00810, FGSG_09280, FGSG_05048, FGSG_02063, FGSG_00175, FGSG_09913, FGSG_03402, FGSG_02482, FGSG_03915, FGSG_01698, FGSG_10200, FGSG_06681, FGSG_05382, FGSG_04196, FGSG_05130, FGSG_06290, FGSG_02974, FGSG_08941, FGSG_11332, FGSG_04194, FGSG_10893, FGSG_03590, FGSG_13714 |
| Cyanoamino acid metabolism | FGSG_00596, FGSG_10659, | FGSG_12231, FGSG_10250, FGSG_06480, FGSG_00313, FGSG_03387, FGSG_01685, FGSG_10859, FGSG_01698, FGSG_09141, FGSG_06290 |
| Butanoate metabolism | FGSG_13963, FGSG_13962, | FGSG_03936, FGSG_05554, FGSG_09494, FGSG_01086, FGSG_06342, FGSG_05128, FGSG_01581, FGSG_00321, FGSG_04103, FGSG_06282, FGSG_00600, FGSG_05048, FGSG_02063, FGSG_04196, FGSG_08941 |
| Aminobenzoate degradation | FGSG_07080, FGSG_07838, FGSG_00489, FGSG_03996, | FGSG_02824, FGSG_04302, FGSG_04736, FGSG_07678, FGSG_03199, FGSG_13176, FGSG_10250, FGSG_13444, FGSG_10120, FGSG_09913, FGSG_03402, FGSG_01698 |
| Bisphenol degradation | FGSG_13962, FGSG_07080, | FGSG_02824, FGSG_04302, |

| | | |
|---|---|---|
| | FGSG_00489, | FGSG_09494, FGSG_04736, FGSG_05128, FGSG_00321, FGSG_10120, FGSG_05048, FGSG_02063, FGSG_09913, FGSG_08941 |
| Ribosome biogenesis in eukaryotes | FGSG_00270, FGSG_02511, FGSG_04424, FGSG_01386, FGSG_00892, FGSG_07262, FGSG_01185, FGSG_07055, FGSG_07258, FGSG_00485, FGSG_06340, FGSG_00732, FGSG_09795, FGSG_05081, FGSG_06070, FGSG_10033, FGSG_06788, FGSG_07206, FGSG_01102, FGSG_10908, FGSG_07379, FGSG_08759, FGSG_10940, FGSG_10219, FGSG_06165, FGSG_01870, | FGSG_03933, FGSG_02500 |
| Methane metabolism | FGSG_07685, FGSG_02217, FGSG_01692, | FGSG_07277, FGSG_00330, FGSG_07127, FGSG_06127, FGSG_02427, FGSG_00475, FGSG_09572, FGSG_00810, FGSG_09280, FGSG_03915, FGSG_10200, FGSG_06681, FGSG_06290, FGSG_02974, FGSG_10893 |
| Glycine, serine and threonine metabolism | FGSG_12344, FGSG_03626, FGSG_07266, FGSG_04468, FGSG_06544, FGSG_01692, FGSG_08407, | FGSG_02357, FGSG_12373, FGSG_08055, FGSG_03638, FGSG_00118, FGSG_02427, FGSG_05623, FGSG_00810, FGSG_10743, FGSG_06290, FGSG_10893 |
| Peroxisome | FGSG_11413, FGSG_02217, FGSG_04333, | FGSG_11069, FGSG_04217, FGSG_05011, FGSG_02287, FGSG_10672, FGSG_07173, FGSG_07104, FGSG_05596, FGSG_06012, FGSG_09424, FGSG_09263, FGSG_10893, FGSG_00840, FGSG_05379, FGSG_09756 |

Table S6. Transcription data for myosin-5, myosin-2B, and type II myosin for strains 2021 and Y2021A of *F. graminearum* in RNA-seq.

| Gene | Description | Length[a] | Fold-change Y2021A/2021[b] | Fold-change 2021+/2021[c] | Fold-change Y2021A+/Y2021A[d] |
|---|---|---|---|---|---|
| FGSG_01410 | Myosin-5 | 3645 | -1.18 | 1.07 | 1.49 |
| FGSG_07469 | Myosin-2B | 4752 | 1.11 | 1.27 | 1.51 |
| FGSG_08719 | Type II myosin | 7029 | -1.29 | 1.15 | 1.72 |

[a] mRNA length in bp.

[b] The fold-change is the ratio of RPKM Y2021A to RPKM 2021.

[c] The fold-change is the ratio of RPKM 2021+ to RPKM 2021. 2021+ represents sensitive strain 2021 treated with 0.21 μg/ml JS399-19 ($EC_{50}$).

[d] The fold-change is the ratio of RPKM Y2021A+ to RPKM Y2021A. Y2021A+ represents resistant strain Y2021A treated with 204 μg/ml JS399-19 ($EC_{50}$).
